# Applying One Health Bioethical Principles to Climate Change: The Planetary Patient Paradigm

**DOI:** 10.7759/cureus.104411

**Published:** 2026-02-27

**Authors:** Zoe Lewczak, Maika G Mitchell

**Affiliations:** 1 Center for Bioethics, Harvard Medical School, Boston, USA; 2 Biotechnology, University of Maryland, Adelphi, USA

**Keywords:** bioethics framework, bioethics recommendations, environmental, global public health, health-care equity, human and animal health, medical school education, one health, one health approach, public and environmental health

## Abstract

Background: Climate change represents a contemporary challenge for global health, requiring frameworks that extend beyond traditional anthropocentric approaches. The One Health paradigm offers a comprehensive framework for addressing these interconnected challenges, yet practical, case-based guidance through bioethical applications remains underexplored.

Objective: To develop and apply a novel analytical framework based on One Health frameworks and bioethical principles for addressing climate change challenges, introducing the ethical metaphor of Earth as a "Planetary Patient" requiring ethical care and treatment.

Methods: We employed a conceptual analysis approach, integrating classical bioethical principles within One Health frameworks. The methodology involved a structured narrative review, principle mapping, case study application, and iterative framework refinement to develop a comprehensive analytical tool for climate health issues.

Results: The theoretical Planetary Patient paradigm provides a structured dimensional approach for evaluating climate interventions: (1) autonomy and self-determination of communities, (2) beneficence and non-maleficence across domains, and (3) justice risk distribution and concepts of intergenerational responsibility. Case study applications demonstrate practical utility across diverse scenarios, including coastal relocation, urban heat mitigation, and agricultural adaptation.

Conclusions: This framework demonstrates how One Health and bioethical principles can be cohesively integrated and systematically applied to the challenges of climate change, offering a practical tool for ethical decision-making in climate adaptation and mitigation efforts. Implementation requires interdisciplinary collaboration and institutional reform but provides significant advantages for comprehensive climate action.

## Introduction

Climate change represents one of the defining health crises of the 21st century, with the World Health Organization projecting an additional 250,000 deaths annually between 2030 and 2050 from malnutrition, malaria, heat stress, and diarrheal disease [1]. The interconnected nature of these fatal threats, spanning human health, animal disease vectors, and ecosystem degradation, demands frameworks that transcend traditional anthropocentric approaches. Recognition of these linkages has evolved considerably, especially in the United States; for instance, the 2009 U.S. Environmental Protection Agency (EPA) Endangerment Findings, under Section 202(a) of the Clean Air Act, officially recognized the link between greenhouse gas emissions and public health, yet today, these findings have faced political and economic scrutiny [2]. The original findings concluded that a combination of six key greenhouse gases poses health and public welfare risks for current and future generations [3]. Crucially, the rule also concluded that the combined emissions of these greenhouse gases from new motor vehicles and new motor vehicle engines contribute to the air pollution that causes this danger. Through scientific evidence and a review of public comments, these findings legally require the EPA to regulate these emissions from motor vehicles [3,4]. However, to better understand the complex interconnectedness of greenhouse gas emissions and public health, one must acknowledge all life systems' interdependence, recognizing the interplay of humans, animals, and the environment [5]. Traditional bioethical approaches, while foundational to medical ethics, may prove inadequate for addressing the robust pluralism posed by climate change across temporal and species boundaries. For instance, because the young field of American bioethics has been dominated by the technological and molecular genetic approaches in biomedicine, bioethicists have cast aside these global public and environmental health challenges [6].

The One Health approach is inherently normative; it suggests a way that the world ought to be and provides a framework for achieving that vision through integrated action across disciplines [7]. From the spread of infectious diseases to the degradation of ecosystems, climate change poses fundamental One Health issues that cannot be adequately addressed through individual, siloed sectors. Consequently, addressing these challenges necessitates a bioethical analysis that extends beyond traditional human-centric concerns to encompass the health and well-being of all life on Earth [5].

This article introduces a conceptual framework that pairs One Health and bioethical principles to address climate change challenges. We propose the ethical metaphor of the "Planetary Patient," a paradigm that conceptualizes Earth and its interconnected systems as a patient in a health crisis, requiring a greater ethical focus on the health of the biosphere [6]. This framework moves beyond viewing Earth merely as a managed resource; rather, we position it as a complex patient, highlighting the need for therapeutic intervention guided by bioethical principles.

Research objectives

The primary objectives of this research are to: (1) develop a comprehensive bioethical approach to integrating One Health with climate action; (2) demonstrate practical application through theoretical case studies; (3) evaluate framework strengths and limitations; and (4) inform discussions for implementation in policy and practice contexts.

## Materials and methods

Conceptual framework development

We developed the Planetary Patient analytical framework through a systematic, multistep approach designed to integrate ethical traditions while maintaining practical applicability.

Literature Analysis

We conducted a comprehensive narrative review of One Health, bioethics, climate change, and environmental ethics literature from 1998 to 2026, sourced from PubMed, Web of Science, and Philosophers' Index databases. The literature review was updated through February 2026 to capture the most recent scholarship on climate adaptation and One Health frameworks to support conceptual analysis.

Principle Mapping

We identified research gaps, then conducted systematic mapping of Beauchamp and Childress’ Principles of Biomedical Ethics, including autonomy, beneficence, non-maleficence, and justice, onto One Health frameworks and environmental ethics approaches [6].

Case Application

We refined and categorized the framework through application to diverse climate health scenarios representing different scales, populations, and ethical challenges. 

Expert Consultation

Iterative development was informed by interdisciplinary expert input from bioethics, public health, veterinary medicine, and environmental science through informal review sessions. 

Framework Validation

We assessed the conceptual framework through the evaluation of strengths, limitations, and practical applicability.

The Planetary Patient concept

The Planetary Patient paradigm has reconceptualized our relationship with Earth’s systems, moving from an extractive, dominion-based approach to one centered on care, stewardship, and healing. This conceptualization draws on the therapeutic relationship model from clinical medicine, positioning humanity and other species as both caregivers and co-patients within a complex, interconnected health system [7].

This paradigm defines climate change not merely as an environmental problem or economic challenge, but as a global health emergency affecting a complex, interconnected patient system requiring coordinated therapeutic intervention [7]. Just as clinical medicine emphasizes the importance of treating the whole patient rather than isolated symptoms, the Planetary Patient approach emphasizes addressing climate change through integrated interventions that consider impacts across all domains of planetary health.

Ethical framework components

Our framework integrates three ethical dimensions adapted from bioethical principles [8,9] but expanded to encompass planetary health considerations.

Autonomy

These dimensions include the recognition of communities’ fundamental right to determine their own adaptation and development pathways, including meaningful participation in decisions affecting their health and environment. This includes respect for Indigenous knowledge systems and traditional practices [5,7,9].

Beneficence and Non-maleficence

The framework focuses on maximizing benefits while minimizing harm across human, animal, and environmental domains [5,8,9]. This requires consideration of both intended and unintended consequences of climate interventions across multiple scales and time horizons.

Justice and Intergenerational Responsibility

It also addresses the equitable distribution of climate risks, benefits, and responsibilities, with particular attention to vulnerable populations, developing nations, and current and future inhabitants [5,8,9]. We have a responsibility to current and future generations as well as long-term ecological integrity, recognizing that current decisions will determine health trajectories for decades or centuries [10]. This includes the preservation of environmental conditions necessary for the health and well-being of humans, animals, and plants.

Analytical methodology

The framework employs a conceptual analytical methodology that evaluates proposed climate interventions through each of the three ethical dimensions. This involves: (1) stakeholder identification and impact assessment across three domains: human, animal, and environmental; (2) temporal analysis considering short-term and long-term consequences; (3) distributive analysis examining how benefits and burdens are allocated; and (4) participatory evaluation ensuring affected communities have meaningful input into decision-making processes.

As this study involves conceptual framework development and theoretical case study analysis without human subjects research, Institutional Review Board (IRB) approval was not required. All case studies presented are hypothetical scenarios constructed for illustrative purposes and were not empirically evaluated.

## Results

Framework application: theoretical climate adaptation case studies

We applied the Planetary Patient framework to four diverse, theoretical climate adaptation scenarios, demonstrating its practical utility across different contexts, scales, and ethical challenges.

Case study 1: coastal community relocation

Scenario

A low-lying Pacific Island community of 2,500 residents faces imminent displacement due to sea-level rise, coastal erosion, and saltwater intrusion affecting freshwater supplies and agricultural land. This illustrative community is likely to contribute less than 1% of global emissions, compared to 14.5% by the United States. Although this is a hypothetical scenario, it is known that Small Island Developing States (SIDS) collectively contribute less than 1% of global emissions yet face disproportionate climate impacts requiring adaptation costs estimated at $50-100 billion annually [11].

Autonomy and Self-Determination Analysis

The framework emphasizes the community’s fundamental right to make informed decisions about its future, including whether, when, and how to relocate. This includes respecting community preferences even when they differ from expert recommendations or sovereign power recommendations. The analysis highlights the critical importance of providing complete information about risks and options while avoiding coercive practices that might pressure communities into specific decisions. This approach necessitates that external agencies support community decision-making capacity rather than imposing predetermined solutions.

Justice and Fairness Analysis

One can estimate that this community contributed minimally to global greenhouse gas emissions, which is about <0.001% of the global total. That said, the principle of distributive justice supports robust international compensation for relocation costs, loss of cultural sites, and disruption of traditional livelihoods. The framework supports the "polluter pays" principle, which recognizes that moral responsibility for damages caused by pollution could be dispersed among the actors who caused the pollution, while recognizing that historical emissions create obligations for high-emitting nations [12]. In essence, those who caused the problem should bear the cost; however, the compensation should address not only economic losses but also cultural and spiritual damages, which may be difficult to quantify.

Implementation Considerations

The framework identified several key implementation requirements: establishing culturally appropriate consultation processes, ensuring community deliberation and decision-making, providing technical and financial support for impact assessment, and developing innovative approaches to maintaining cultural identity and community cohesion during relocation processes.

Case study 2: urban heat island mitigation

Scenario

A major metropolitan area plans comprehensive urban forest planting and green infrastructure development in low-income neighborhoods experiencing disproportionate heat exposure and related health impacts. Studies of urban greening in U.S. cities have documented property value increases of 5-15% within two years of nearby park development, which is associated with 10-30% displacement of the original low-income residents in unprotected neighborhoods [13]. Portland, Oregon’s inclusionary zoning policies paired with green space development offer a model for preventing such displacement [14].

Beneficence Analysis

The intervention provides clear benefits for human health through ambient temperature reduction, which is estimated to be 2-5°C cooling. This improves air quality through pollutant removal, and the abundance of green space leads to enhanced benefits for physical and mental health [15]. The environmental benefits include habitat creation for urban wildlife, carbon sequestration, stormwater management, and biodiversity enhancement. The framework supports interventions that provide co-benefits across human, animal, and environmental domains.

Non-maleficence Analysis

The framework identified potential harms requiring mitigation: possible green gentrification leading to displacement of existing residents as property values increase; allergens from certain tree species; increased maintenance costs potentially burdening municipal budgets; and unintended consequences such as pest habitat creation or root damage to infrastructure. It is important to recognize that the design and maintenance of accessibility and attractiveness of these green spaces play a major role in their usage [15]. Ethical implementation requires proactive measures to prevent these harms while maximizing benefits. For instance, if a green space is not well-maintained or poorly lit, it leads to an opportunity for the space to be underutilized.

Mitigation Strategies

The analysis recommended several harm reduction approaches: selection of hypoallergenic native species, implementation of anti-displacement policies including affordable housing preservation, community ownership models for green infrastructure, and comprehensive monitoring of both benefits and potential negative impacts. There is also potential for establishing rental programs for community events and social activities to ensure the space remains a vibrant neighborhood hub rather than an underutilized area. This also provides a local revenue stream to offset ongoing maintenance costs.

Case study 3: agricultural adaptation policies

Scenario

National development of climate-resilient crop varieties and farming practices to address changing precipitation patterns, rising temperatures, and the increased frequency of extreme weather events affecting food security.

Intergenerational Responsibility Analysis

The framework emphasizes developing agricultural practices that maintain productivity for current generations while preserving soil health, water resources, and genetic diversity for future generations, further supporting the idea of intergenerational equity [10]. Our responsibility is to hand the Earth over to the next generation in as good a condition as we found it. We should support sustainable intensification approaches that increase yields without compromising long-term productive capacity. The analysis highlighted the importance of preserving traditional crop varieties and Indigenous agricultural knowledge as resources for future adaptation [16].

Autonomy Analysis

The framework insists on participatory approaches that incorporate farmers’ Indigenous knowledge, preferences, and local conditions rather than imposing external solutions. This includes respecting farmers’ rights to save and exchange seeds, maintain traditional practices, and make independent decisions about technology adoption. There should also be consideration for extension services, which support farmer decision-making rather than dictating specific approaches [10,16].

Long-Term Sustainability Measures

The framework supports policies that incentivize practices benefiting long-term sustainability: crop rotation systems that maintain soil health, integrated pest management reducing chemical inputs, agroforestry approaches providing multiple benefits, and water conservation techniques appropriate for changing precipitation patterns [16]. These approaches require initial investments but provide long-term benefits for both productivity and environmental health.

Case study 4: sustainable data center integration

Scenario

A tech firm proposes a large-scale data center in an urban-fringe neighborhood. While it brings digital infrastructure and tax revenue, the community is concerned about energy and water consumption and "dead space" that provides no social value to residents. Notably, there is an increased demand for generative artificial intelligence (AI) models, which necessitates extensive use of data storage with compound energy-water-climate impacts. It is estimated that the use of AI servers in the United States could generate an annual water footprint ranging from 731 to 1,125 million m^3^ and carbon emissions ranging from 24 to 44 Mt CO_2_-equivalent each year [17].

Beneficence Analysis

It is important to understand the technical and economic benefits of such a project. For instance, waste heat recovery can capture excess heat from servers to provide low-cost district heating for local homes, while the economic impact results in local tax revenue and job creation [18]. Furthermore, data centers offer benefits in the form of enhanced digital usage and infrastructure for the region.

Non-maleficence Analysis

The framework identifies several potential harms requiring mitigation. There is a significant risk of resource strain, as high electricity and water usage may impact local utility costs, drain water supplies, and cause ecological damage [19]. Additionally, data centers are often criticized for their unattractive, warehouse-like structures that can create a dead zone in the neighborhood, leading to social exclusion and underutilized urban space [20]. Beyond human impact, the project poses risks to local fauna through noise pollution from cooling fans, which can disrupt avian communication, and light pollution, which affects the migratory patterns of nocturnal species. Additionally, the physical footprint can lead to habitat fragmentation, creating "dead zones" that isolate local animal populations [21].

Mitigation Strategies

The "Community Hub" model: Because data centers are often seen as land-hungry users that do not always give back to neighbors, a Community Hub Model is proposed. Rather than a closed fortress, the facility should incorporate public-facing design, including a Green Wing with public-access gardens and meeting rooms. To ensure the building serves as a vibrant neighborhood hub, the plan establishes community social programming by offering rental community spaces for events, workshops, and social activities.

Following the "polluter pays" principle, the developer provides compensation for the resource-heavy nature of the facility by paying for local infrastructure upgrades, supporting human and non-human well-being [12]. Furthermore, rental income from the community spaces is directly earmarked for revenue reinvestment to fund local mitigation, such as the planting of native species discussed in previous scenarios, in an agreement to help offset the net negatives of these facilities.

Framework validation and assessment of the approach’s strengths

This theoretical Planetary Patient framework demonstrates several unique strategies over traditional climate policy approaches. By providing a holistic integration of human, animal, and environmental domains within a coherent ethical structure, this systematic approach avoids the fragmentation typical of sectoral policies, ensuring that the well-being of all sentient beings, including future generations, is addressed [5,10]. Practical applicability is evidenced through case-study simulations, which demonstrate how established bioethical principles can provide concrete guidance for complex projects, such as sustainable data center integration or urban forest development. By emphasizing autonomy and participation, the framework ensures cultural adaptability and democratic legitimacy, fostering community ownership over interventions like coastal relocation or neighborhood greening. Furthermore, its commitment to temporal comprehensiveness directly tackles intergenerational responsibility, treating current populations as trustees rather than owners of the planet’s heritage [10]. Ultimately, this framework provides the ethical rigor necessary to maintain practical relevance while ensuring that today’s policy decisions do not discount the needs of those heirs who will inherit our environmental legacy.

Limitations and challenges

Drawing from the framework applications, several significant limitations and implementation challenges have been identified. Managing the inherent complexity of the "Planetary Patient" model can lead to analysis paralysis, as balancing multiple ethical considerations simultaneously may result in conflicting courses of action. A primary difficulty lies in value quantification, particularly when attempting to compare intangible or intrinsic ethical values that resist traditional cost-benefit analysis [5]. Furthermore, the framework’s high resource intensity requires substantial investments in stakeholder engagement, interdisciplinary collaboration, and comprehensive impact assessments that may exceed the capacity of available resources.

Beyond resource needs, institutional barriers present a major hurdle, as implementation necessitates significant changes to existing structures, professional training programs, and established policymaking processes. These requirements also create significant time constraints, making it challenging to apply the full comprehensive analysis in emergencies that demand a rapid response [5,7]. Finally, scale coordination remains a persistent issue, as it is difficult to align ethical analysis across local, national, and global levels when each tier possesses varying priorities and capacities.

Comparative analysis

Comparison with alternative approaches to climate policy reveals distinct advantages and trade-offs. Economic cost-benefit analysis provides quantitative precision but fails to address distributional concerns and non-market values [5]. Purely technological approaches may achieve efficiency gains but neglect social and ethical dimensions. Traditional environmental impact assessment addresses some environmental concerns but typically maintains anthropocentric perspectives [22]. As shown in Table 1, a consolidated overview of alternative approaches to climate policy highlights the distinct advantages and trade-offs inherent in each strategy [4,5,7-10].

**Table 1:** Comparing Alternative Approaches to Climate Policy EIA, environmental impact assessment

Approach	Strengths	Limitations	Best Use Case
Planetary Patient	Holistic, ethical integration	Resource-intensive, complex	Complex, high-stakes, long-term
Cost-benefit analysis	Quantifiable, efficient	Ignores equity, non-market values	Short-term economic decisions
Rights-based	Clear moral foundations	Rigid, competing claims	Individual protections
Traditional EIA	Established process	Anthropocentric, limited scope	Regulatory compliance

The Planetary Patient framework offers superior integration of ethical considerations but requires greater resource investments and institutional coordination compared to simpler approaches. The framework is most valuable for complex, high-stakes decisions with significant distributional implications and long-term consequences.

## Discussion

Implications for health professionals

Healthcare clinicians and public health practitioners occupy a unique position to implement the Planetary Patient framework. As trusted community figures, clinicians can advocate for climate-health interventions that align with bioethical principles familiar from clinical practice. Medical and public health schools are increasingly endorsing One Health into curricula [7], creating opportunities for workforce development. Public health departments can adopt the framework for climate resilience planning, ensuring that adaptation measures protect vulnerable populations through autonomy-respecting community engagement and equitable resource distribution.

Ethical implications of the Planetary Patient paradigm

Clinicians and public health practitioners occupy a unique position to implement the Planetary Patient framework. As trusted community figures, clinicians have the potential to be advocates for climate-health interventions that align with bioethical principles familiar from clinical practice. Medical schools increasingly integrate planetary health into curricula [7], creating opportunities for workforce development. Public health departments can adopt the framework for climate resilience planning, ensuring that adaptation measures protect vulnerable populations through autonomy-respecting community engagement and equitable resource distribution.

The Planetary Patient concept represents a significant reconceptualization of human-environment relationships with profound implications for climate policy and practice. By framing Earth as a patient requiring care rather than a resource to be exploited, this paradigm shifts attention from extraction and dominion toward healing and stewardship [5,7]. This reconceptualization challenges anthropocentric assumptions underlying much current climate policy while providing practical guidance for more ethical approaches.

The therapeutic relationship model emphasizes the importance of informed consent, shared decision-making, and ongoing care relationships rather than one-time interventions. Applied to climate action, this suggests the need for sustained engagement with affected communities, adaptive management approaches that can respond to changing conditions, and recognition that climate interventions require ongoing monitoring and adjustment rather than simple implementation of predetermined solutions [3,4].

The framework’s expansion of ethical consideration beyond human interests raises important questions about the moral status of non-human species and ecological systems. While not requiring specific philosophical commitments, such as those embedded in animal rights or environmental ethics arguments, the framework does require systematic consideration of impacts on all components of planetary health systems, including but not limited to the sole anthropological pieces.

Practical implementation considerations

Successful implementation of the Planetary Patient framework necessitates a shift in institutional structures, professional practices, and policy processes. A foundational requirement is the development of interdisciplinary education programs that merge bioethics, environmental science, public health, and social sciences, equipping professionals for collaboration across traditional disciplinary boundaries [5,7,23]. Beyond education, ethical analysis must be formally integrated into policy development, climate-adaptation planning, and environmental impact assessments at local, national, and international levels. This transition also requires a robust community-engagement infrastructure, establishing mechanisms for meaningful participation that include capacity building for affected populations and culturally appropriate consultation processes [23].

To ensure long-term efficacy, the framework calls for sophisticated monitoring and evaluation systems using indicators that assess ethical outcomes across human, animal, and environmental domains, thereby enabling accountability and adaptive management [5]. These efforts must be supported by systemic institutional reform, including changes to funding mechanisms, organizational structures, and professional incentives that currently hinder integrated approaches to climate challenges [24]. Ultimately, this implementation should be gradual and adaptive, beginning with focused pilot applications to allow for iterative refinements and the development of best practices before scaling to broader policy domains.

Comparison with alternative approaches

The Planetary Patient framework offers unique solutions compared to alternative approaches to climate ethics and policy. Compared to purely consequentialist approaches such as cost-benefit analysis, the framework provides better attention to distributive concerns and non-quantifiable values. Rights-based approaches are often too rigid and cannot account for nuanced details. This framework offers greater flexibility and practical guidance for complex situations involving competing claims [5,10]. While traditional environmental impact assessments have dominated these arguments for decades, this framework provides more systematic attention to ethics and broader consideration of impacts across species and generations; it also provides theoretical grounding and a systematic methodology while maintaining a commitment to democratic engagement [10,23]. However, the framework also has limitations compared to some simpler approaches. It requires more integrated resources and time than streamlined decision-making processes, may be challenging to implement in emergencies, and demands specialized expertise that may not be readily available in all contexts. The choice of approach should depend on the stakes involved, available resources, and the complexity of ethical trade-offs requiring consideration.

## Conclusions

This theoretical model of the Planetary Patient conceptualization provides a powerful metaphor for reconceptualizing One Health through care and stewardship lenses. The framework demonstrates how established bioethical principles can be systematically extended to address the complex, interconnected health challenges posed by climate change across species and temporal boundaries. Through detailed case-study applications, we have shown that the framework provides practical guidance for ethical decision-making across diverse climate-adaptation and mitigation contexts. The case-study applications demonstrate the framework’s practical utility while also revealing implementation challenges that must be addressed for broader adoption. While this framework requires significant investments in interdisciplinary collaboration, stakeholder engagement, and institutional reform, it offers a superior integration of ethical considerations compared to alternative approaches. The complexity of ethical analysis may be challenging in some contexts, but it is justified by the magnitude and complexity of climate change impacts. Future research should focus on developing practical implementation tools and evaluating framework effectiveness in diverse real-world applications while addressing remaining theoretical challenges.

As climate change impacts continue to intensify and their effects on planetary health become increasingly severe, we ought to shift our thinking on this topic. The change is now: we need a comprehensive, ethically grounded approach to climate action that will only become more urgent. The Planetary Patient paradigm provides a vital framework for meeting this challenge, offering a systematic approach to climate ethics that is both theoretically rigorous and practically applicable. In treating the planet as our patient, we honor a partnership spanning generations, ensuring justice for the living, while the yet-to-be-born inherit a world of flourishing options rather than a legacy of our debts.
